# Rapid High Resolution Genotyping of *Francisella tularensis* by Whole Genome Sequence Comparison of Annotated Genes (“MLST^+^”)

**DOI:** 10.1371/journal.pone.0123298

**Published:** 2015-04-09

**Authors:** Markus H. Antwerpen, Karola Prior, Alexander Mellmann, Sebastian Höppner, Wolf D. Splettstoesser, Dag Harmsen

**Affiliations:** 1 Bundeswehr, Institute of Microbiology, Neuherbergstrasse 12, 80937, Muenchen, Germany; 2 University of Muenster, Department of Periodontology, Waldeyerstrasse 30, 48149, Muenster, Germany; 3 University of Muenster, Institute of Hygiene, Robert-Koch-Strasse 41, 48149, Muenster, Germany; 4 Rostock University Hospital, Institute of Medical Microbiology, Virology and Hygiene, Schillingallee 70, 18057 Rostock, Germany; Institut National de la Recherche Agronomique, FRANCE

## Abstract

The zoonotic disease tularemia is caused by the bacterium *Francisella tularensis*. This pathogen is considered as a category A select agent with potential to be misused in bioterrorism. Molecular typing based on DNA-sequence like canSNP-typing or MLVA has become the accepted standard for this organism. Due to the organism’s highly clonal nature, the current typing methods have reached their limit of discrimination for classifying closely related subpopulations within the subspecies *F*. *tularensis* ssp. *holarctica*. We introduce a new gene-by-gene approach, MLST^+^, based on whole genome data of 15 sequenced *F*. *tularensis* ssp. *holarctica* strains and apply this approach to investigate an epidemic of lethal tularemia among non-human primates in two animal facilities in Germany. Due to the high resolution of MLST^+^ we are able to demonstrate that three independent clones of this highly infectious pathogen were responsible for these spatially and temporally restricted outbreaks.

## Introduction

Rapid and cost-effective generation of whole-genome sequence (WGS) data for bacterial pathogens is currently revolutionizing our understanding of the taxonomy, phylogeny, genomic diversity and population dynamics of several clinically relevant microorganisms [1, 2]. Sequence-based DNA signatures are increasingly used for molecular typing and reliable classification of bacterial isolates or pathogen-related DNA samples extracted directly from clinical specimens [3, 4]. Nucleotide sequence-based typing methods provide unambiguous results which can be easily stored, distributed and compared between laboratories. New developments in sequencing technologies and namely “next-generation sequencing” approaches now allow for rapid and inexpensive sequencing of draft genomes of pathogenic bacteria of numerous genera [5]. Whole genome comparisons reveal a plethora of genetic polymorphisms which can be used for fast and accurate classification of disease isolates down to the single strain level. Reliable genotyping is a prerequisite for identifying the infectious agent and thus for diagnosing an infectious disease [i], for outbreak investigations and describing natural transmission patterns [ii], for phylogeographic studies delineating spatial and temporal distribution of bacterial pathogens [iii], and for comprehensively analyzing population dynamics aimed at rapidly identifying new or emerging variants of the biological agent with altered virulence or resistance characteristics [iv] [1].

Fulfilling these major tasks is especially important but also challenging for a highly infectious and genetically monomorphic pathogen like *Francisella tularensis* [reviewed by [6]]. The Gram-negative bacterium is the causative agent of tularemia, a zoonotic disease widespread over the Northern Hemisphere. Its transmission is complex and involves numerous mammal hosts and arthropod vectors [7]. In humans the clinical picture varies from skin ulceration to severe pneumonia or septicemia with fatal outcome depending on the route of infection and subspecies causing the disease [8]. The low infectious dose of 10–50 CFU in aerosols combined with its potential lethality if untreated, were reasons for considering this organism in former bioweapon programs [9]. Thus, *F*. *tularensis* is listed as a category A select agent according to the Centers for Disease Control and Prevention [CDC] [10].

A hierarchical approach for genotyping this bacterium comprises a two-step-procedure: determining canonical single nucleotide polymorphisms [canSNPs] and/or insertion and deletions [INDELS] and multi locus variable number of tandem repeats analysis [MLVA] aided in dissecting the phylogenetic relationship between and among existing subspecies. It also revealed the current evolution of *F*. *tularensis*, which is characterized by the ongoing global expansion of a highly competitive clone of *F*. *tularensis* ssp. *holarctica* [11].

One alternative approach which has been successfully employed for cataloguing bacterial diversity in the WGS era is a MLST-like gene-by-gene approach built on *de novo* assembly of the sequenced bacterial genome followed by annotation and cumulative comparison of defined genetic elements [5]. This gene-by-gene [MLST^+^] approach allows the scalable organization and adaption of the typing data according to clinical or scientific needs. While this effort has been shown to be successful in evaluating the epidemiology of *Campylobacter*, *E*. *coli* O104 or methicillin-resistant *Staphylococcus aureus* [MRSA] in public health surveillance or resolution of a meningococcal disease outbreak, such approaches remain to be established for species with a highly monomorphic or clonal population structure [4, 5, 12]. Unfortunately, traditional MLST does not provide sufficient discrimination power for resolving differences among single strains of monomorphic, low diversity, asexual pathogens such as *F*. *tularensis*, *Yersinia pestis* or *Bacillus anthracis* [13].

To tackle the emerging challenge of limited genetic resolution power of established typing markers [canSNP, VNTRs or MLVA], we present a high-resolution genotyping approach for outbreak strains using MLST^+^, a combination of whole genome sequencing and gene-by-gene comparison. First, we sequenced type strain *F*. *tularensis* ssp. *holarctica* LVS using IonTorrent PGM and Illumina MiSeq to exclude any technology-introduced bias of MLST^+^ during data evaluation. Secondly, we investigated samples collected from two animal facilities during a period from 2002 to 2006 [14, 15] and performed MLST^+^ analysis after *de novo* assembly using the software package SeqSphere^+^. We compared our results with the current “gold-standard” approach comprising canSNP and MLVA typing. Here, we demonstrate that the MLST^+^ approach was superior to the present standard. By this procedure we elucidated that the investigated outbreaks were caused by three different clones of *F*. *tularensis* spp. *holarctica*.

## Material and Methods

### Strains and DNA Preparation

One clinical isolate from a bacteremic patient [F233] and 13 outbreak isolates of the *Francisella* strain collection of the German reference laboratory for tularemia were selected for whole genome sequencing [Table 1]. Strain F233 was isolated from a tularemia patient living on Western Germany in 2010. The outbreak isolates comprised i] five strains isolated from different organs of *Macacae* housed in the German Primate Center, Goettingen, one strain [F107] was isolated in 2005, the remaining four strains [F100, F101, F108, F109] were isolated from animals who succumbed to tularemia infection in 2002 [14]; ii] seven strains isolated from marmosets [*Callithrix jacchus*] which were housed in a separate animal facility in Sennickerode, county of Goettingen in the years 2004 [F88, F89, F90, F91, F92] and 2006 [F112, F114] [15]; iii] one strain isolated from two water voles [*Arvicola terrestris]* which were trapped in 2005 during an epidemiological investigation at the facility [F105] [16]. For determining of technological bias, strain LVS [NC_007880.1] was chosen, as the finished genome sequence of this standard laboratory strain was publically available.

**Table 1:** Sequenced strains of this study with results of canSNP-, MLVA-, and MLST^+^-Analysis and ENA-Accession numbers.

Strain	Host Species	Year	Origin	canSNP Cluster	MLVA − Genoytype	MLST + Genotype	ENA AccNo
F088	*Callithrix jacchus*	2004	Sennickerode	B.Br.FTNF002-00	1	1	ERS483168
F089	*Callithrix jacchus*	2004	Sennickerode	B.Br.FTNF002-00	1	1	ERS483169
F090	*Callithrix jacchus*	2004	Sennickerode	B.Br.FTNF002-00	1	1	ERS483166
F091	*Callithrix jacchus*	2004	Sennickerode	B.Br.FTNF002-00	1	1	ERS483167
F092	*Callithrix jacchus*	2004	Sennickerode	B.Br.FTNF002-00	1	1	ERS433995
F105	*Arvicola terrestris*	2005	Sennickerode	B.Br.FTNF002-00	1	1	ERS403447
F112	*Callithrix jacchus*	2006	Sennickerode	B.Br.FTNF002-00	1	1	ERS403448
F114	*Callithrix jacchus*	2006	Sennickerode	B.Br.FTNF002-00	1	1	ERS421610
F233	*Homo sapiens*	2010	Reipoltskirchen	B.Br.FTNF002-00	1	2	ERS483170
F108	*Macaca fascicularis*	2002	Göttingen	B.Br.FTNF002-00	2	3	ERS485186
F109	*Macaca fascicularis*	2002	Göttingen	B.Br.FTNF002-00	2	3	ERS403300
F100	*Macaca fascicularis*	2002	Göttingen	B.Br.FTNF002-00	2	3	ERS396003
F101	*Macaca fascicularis*	2002	Göttingen	B.Br.013/014 B.Br.034/035	3	4	ERS401474
F107	*Macaca silenus*	2005	Göttingen	B.Br.013/014 B.Br.034/035	3	4	ERS403299

All *Francisella tularensis* strains [Table 1] were grown on Heart-Cysteine Agar at 37°C and 5% CO_2_ for 48 hours. DNA preparation from bacterial culture was performed using the Qiagen Genomic Tip Kit [Qiagen, Hilden, Germany] according to the manufacturer´s protocol. Purified DNA was dissolved in PCR-grade water. DNA purity and integrity was checked by gel-electrophoresis. DNA concentrations were determined using the Qubit dsDNA BR Assay [Life Technologies, Darmstadt, Germany].

### Phylogenetic Analyses using canSNPs and Multi Locus VNTR Analysis [MLVA]

Determination of canSNPs status and assignment of strains to different phylogenetic branches were done according to [17]. For differentiation within subclades, MLVA was performed according to [18], using an additional twelfth marker Ft-M26 [17]. Fragment length determination was performed on the capillary sequencer ABI Genetic Analyzer 3100 using GeneScan 1200 LIZ Size Standard [both Life Technologies]. Fragment lengths were calibrated to *in silico* data of *F*. *tularensis* ssp. *holarctica* strain LVS and *F*. *tularensis* ssp. *tularensis* strain SchuS4.

### Whole Genome Sequencing and MLST^+^ Analysis

Prior to genome sequencing, library preparation using 1 μg extracted DNA was performed with the Ion Xpress Fragment Library Kit comprising the Ion Shear-chemistry [Life Technologies] in combination with the Ion Xpress Barcode Kit [Life Technologies] enabling for multiplexing. The E-Gel system was used for size selection as described in the user guide. All steps were quality controlled by Caliper LabChip GX using the HT DNA High Sensitivity Assay Kit [Caliper Life Science, Mainz, Germany]. Up to four libraries were pooled per sequencing run. Template preparation was performed with the One Touch 2 instrument and the Ion PGM Template OT2 200 Kit [Life Technologies] according to the manufacturer’s instructions. Amplification quality was estimated on the Guava easyCyte5 system [Millipore, Schwalbach, Germany]. For each sequencing run one Ion 318 chip was loaded and subsequently sequenced on an Ion Torrent PGM benchtop sequencer [Life Technologies] using 640 run flows—resulting in an average reading length of 300 nucleotides—according to the Ion PGM Sequencing 300 Kit [Life Technologies], user guide. Adjusted stringency-parameters [q15w10] were used to obtain only high accuracy data. De-pooling of obtained reads was performed using the integrated software evaluation pipeline. Gained reads were downsampled using in-house Python-Scripts to an estimated 40-fold coverage.

For sequencing using Illumina MiSeq [Illumina, San Diego, US], the purified genomic DNA was quantified with Qubit dsDNA BR Assay Kit [Life Technologies] and normalized to 2 ng/μL. The MiSeq library was prepared with the Nextera DNA Sample Preparation Kit [Illumina] according to the manufacturer’s instructions using 50 ng of DNA and adapters P7 and P5 with indices for multiplexing purposes. Size-estimation of the library was performed on a Caliper LabChip GX as described for Ion Torrent PGM-Sequencing. Library-concentration was determined with the Qubit dsDNA HS Assay Kit [Life Technologies]. The library was diluted to 2 nM in molecular biology grade water and a pool with an appropriate amount of other libraries was prepared. The estimated final coverage should be about 70fold for each pooled strain. The library pool was further diluted to the working concentration [10 pM] and spiked with PhiX-control as described in the user guide. The MiSeq run was performed with the MiSeq Reagent Kit [500-cycles PE, Illumina] following the standard protocol for Nextera libraries with 2x251 cycles and two times 8 additional cycles for the barcodes using the MiSeq Control Software v2.0.5. FASTQ files were created using the MiSeq Reporter Software [MRS] with default settings.

*De novo* assembly was performed from obtained FASTQ files using MIRA 3.9.9 [19] with default settings and one adjustment for PGM data assembly:-Assembly:mrpc = 10. Alignments were stored as ace-files for further analysis.

### Confirmation of Differences within Genomic Clusters

Sanger sequencing was used to confirm differences within genomic clusters determined by Ion Torrent WGS. For targets of interest individual sequencing primers were designed according to routine protocols.

### MLST^+^ Based Analysis Pipeline

A MLST^+^ scheme was defined using the MLST^+^ Target Definer tool of the Ridom SeqSphere^+^ version 2.3.1 software [Ridom GmbH, Münster, Germany] with default settings. The finished genome of the *F*. *tularensis holarctica* strain LVS [GenBank ID NC_007880.1] served as reference genome [1754 genes]. Default settings comprise the following filters for genes of the LVS reference genome that are excluded from the MLST+ scheme: a “minimum length filter” that discards all genes shorter than 50 bp; a “start codon filter” that discards all genes that contain no start codon at the beginning of the gene; a “stop codon filter” that discards all genes that contain no stop codon, more than one stop codon or if the stop codon is not at the end of the gene; a “homologous gene filter” that discards all genes with fragments that occur in multiple copies within a genome (with identity 90% and more >100bp overlap) and a “gene overlap filter” that discards the shorter gene from MLST+ scheme if the affected two genes overlap >4 bp. The remaining genes were then used in a pairwise comparison using BLAST version 2.2.12 with the query genomes. The following twelve query genomes were used: *F*. *tularensis* ssp. *tularensis* [strain SchuS4, NC_006570.2; strain TIGB03, NC_016933.1; strain WY96-3418, NC_009257.1, strain TI0902, NC_016937.1, strain NE061518, NC_017453.1, strain FSC198, NC_008245.1], *F*. *tularensis* ssp. *holarctica* [strain FTNF002-00, NC_009749.1, strain FSC200, NC_019551.1, strain OSU18, BK006741.1], *F*. *tularensis* ssp. *mediasiatica* [strain FSC147, NC_010677.1], and *F*. *novicida* [strain FX1, NC_017450.1; strain U112, NC_008601.1]. All genes of the reference genome that were common in all query genomes with a sequence identity ≥90% and 100% overlap, and with the default parameter “stop codon percentage filter” turned on (this discards all genes that have internal stop codons in more than 20% of the query genomes) formed the final MLST+ scheme. Finally, assembled reads [ace-files] of all sequenced strains were imported and analysed by applying the default quality criteria [e.g. no frame shift allowed in genes] and with the software’s auto-correction for homopolymer related insertion and deletion errors turned on. This correction takes into consideration the length of homopolymers, absolute read coverage, percentage of reads matching the reference sequence, and strand information. Allele assignment for the previously defined scheme genes was performed to those loci present in the analysed genome to obtain a strain specific allele profile.

Moreover SeqSphere+ was used to conduct a core genome gene SNP analysis with default settings. For better comparability with the MLST+ allelic profiles the conservative approach, i.e. all targets with missing values in one of the strains compared were removed from the analysis, was chosen. With this approach nucleotides from positions responsible for allele differentiation were extracted from all investigated strains and aligned. Minimum spanning trees [MST] were drawn with SeqSphere+ from the allelic profiles and the core genome gene SNP analysis to visualize strain relationships. For calculation of the MLST^+^-MST a conservative approach was chosen: targets not present in one of the strains compared were omitted from further calculation. When comparing different strain-subsets this may lead to different total numbers of valid targets. Using software-package MEGA5 [20] cladograms for SNP-analysis and MLST^+^ allelic profiles were calculated using a maximum parsimony-algorithm. The UPGMA-algorithm was employed for analysis of MLVA data. Bootstrap analysis was performed for both methods with 1000 iterations.

## Results

### Development of a MLST^+^ Scheme

To allow standardized genome based genotyping of clinically lethal *F*. *tularensis* isolates, we developed a MLST^+^ based genotyping scheme. Using *F*. *tularensis* ssp. *tularensis* strain LVS [GenBank ID NC_00962.3] as reference genome [1754 targets] and further twelve genomes *F*. *tularensis* strains as query genomes, we defined a standard set of 1147 targets [65.4% of the reference genome targets] for the MLST^+^ scheme. Included and excluded targets are listed in technical appendix. Excluded targets may be implemented and handled during subsequent analysis as accessory genes, but were neglected in this study to follow a conservative comparison strategy. After saving the extracted sequences of the target genes into the nomenclature database, assembled whole genome data of 14 isolates were compared to each other as described below.

Strain LVS was sequenced twice by using the Illumina MiSeq and IonTorrent PGM technologies, respectively. Each result was compared to the published RefSeq-genome for the defined core genome consisting of 1147 targets. A high recovery rate [PGM 1142/1147; MiSeq 1146/1147] was observed for both technologies and the allelic pattern of 1142 genes was identical with both approaches. One difference to the genome [RefSeq: NC_007880] published in 2006, was identified in one gene [FTL_0146, ABC transporter ATP-binding protein] with both technologies. Here, Sanger sequencing results corroborated a nucleotide substitution G → T at position 1022.

### MLST^+^ Analysis of Outbreak Strains

**Fig 1:**
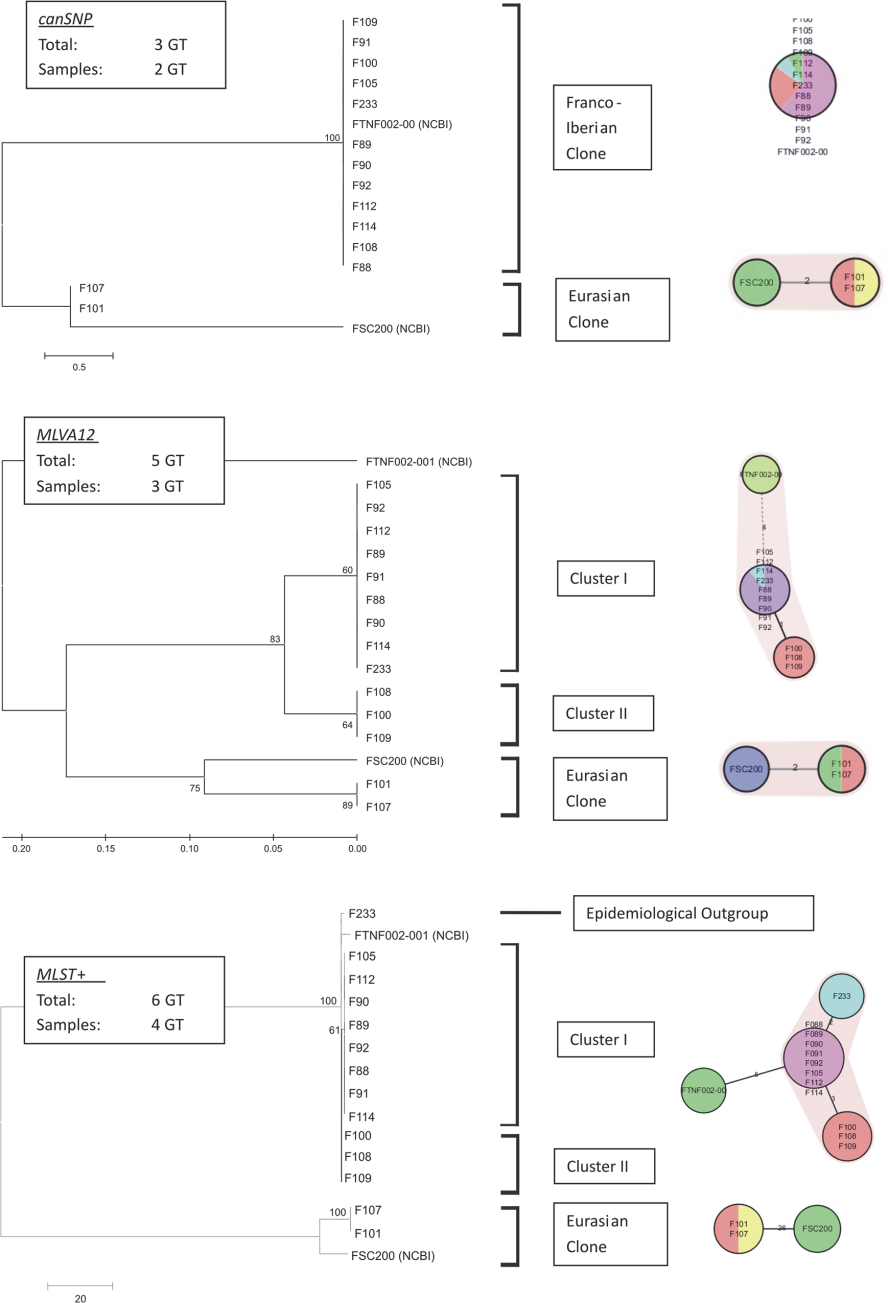
Comparison of results different typing systems. Cladogram and minimum spanning tree depicting relationship among sequenced isolates obtained by bootstrap-analysis [1000 repeats]. Top: canSNP-Typing [maximum parsimony-cladogram], Middle: MLVA-Typing [UPGMA-cladogram], Bottom: MLST^+^-Typing [maximum parsimony cladogram].

All newly generated draft whole genome data were downsampled to 40-fold coverage before performing the *de novo* assembly. The average read-length was 203bp. The genomes showed a recovery rate of core genome targets varying from 97.65% [1123 targets] to 99.91% [1146 targets] with a mean of 98.92% [1137 targets] of the 14 strains. Next a MLST^+^ MST analysis with the in-house-sequenced isolates and NCBI RefSeq genomes was performed. Two main clusters were identified with prominent isolates *F*. *tularensis* ssp. *holarctica* strains FTNF002 [GenBank Acc No: NC_009749.1] and FSC200 [NCBI SRA Archive SRR518502] [Fig 1]. Both strains are the genetically closest reference strains currently available to the investigated outbreak strains and belong to the Eurasian-cluster [11] [strain FSC200], which is present in middle-eastern Europe, and the Franco-Iberian cluster [strain FTNF002] that is dominant in Western Europe.

Strains isolated 2002 [F101] and 2005 [F107] in Goettingen cluster to the Eurasian-group. Comparison of only FSC200 with these isolates showed 26 differences in the allelic-pattern of 1142 target genes.

The remaining isolates group with reference strain FTNF002 into the Franco-Iberian cluster. Within this cluster MLST^+^ can distinguish between three different genotypes: i] cluster I [8 strains] isolated in Sennickerode from 2004 to 2006; ii] cluster II [F100, F108, F109] comprising three strains isolated only in 2002 in Goettingen and iii] the human isolate [F233] isolated in 2010 in Reipoltskirchen. A MST of 1100 target genes shows 2 allele-differences in patterns compared F233 to cluster I with eight strains, that are based on the following point mutations: FTL_1282 [beta glucosidase] 5C→A and FTL_1534 [uridine kinase] 648T→C. Cluster II features three differences compared to cluster I: FTL_0708 [hypothetical protein] 133G→A, FTL_1218 [hypothetical protein] 663C→ A and FTL_1534 [uridine kinase] 648T→C. Strain FTNF002 differs to cluster I in 5 targets. [see details in S1 Data Archive] Sanger sequencing could confirm all nucleotide substitutions between the three clusters.

Therefore, MLST^+^ can differentiate between three genotypes present in the investigated outbreak strain collection comprising isolates sampled within an area of 20 km^2^. In year 2002 Eurasian and Franco-Iberian clones were found in Goettingen, whereas in year 2005 only the Eurasian clone could be identified. In Sennickerode, 20 km apart from Goettingen, only Franco-Iberian clones were identified, independent of host-species or year of isolation. However, within this clone, isolates from Goettingen and Sennickerode could be distinguished by different MLST^+^-genotypes. A fourth genotype was observed for the human isolate F233 originating from Reipoltskirchen, located 260 km west of Sennickerode

Core genome gene SNP comparison did not show any additional information for intra-cluster comparison, based on the fact, that only one nucleotide-position was responsible for assigning a new allele. However, a higher amount of differences could be observed between the Franco-Iberian group and the F. tularensis ssp. holarctica LVS-strain (341 vs. 269) (see S1 Data Archive)

### CanSNP and MLVA Determination

The hierarchical approach of canSNP- and MLVA analysis is the current gold-standard of epidemiological investigations of *F*. *tularensis*. Determination of canSNPs [11] revealed two different branches in our collection of 14 isolates: B.Br.013/014 [Eurasian-cluster; [2 isolates; F101, F107]] and B.Br.FTNF002-00 [Franco-Iberian-cluster]. Further analysis using additional SNP-markers[21] allowed the sub-classification of the Eurasian-isolates to sub-branch B.Br.034/035 in strains from Goettingen between 2002 or 2005. The second branch with 12 isolates belonged to the Franco-Iberian-cluster. This group comprises all isolates from Sennickerode, three isolates [F100, F108, F109] from Goettingen and the human isolate [F233] from Reipoltskirchen.

Next, we used MLVA to determine its discriminatory power for this strain collection. MLVA identified three different lineages: The Eurasian lineage with two strains [F101, F107] and two different lineages within the Franco-Iberian-cluster. Within this Franco-Iberian-cluster strains from Goettingen showed a pattern different from those isolates near Sennickerode. For this marker, Ft-M03 from Sennickerode contained one repeat more than Goettingen-strains [312 bp vs. 303 bp fragment length] [see S1 Data Archive]. The human isolate F233 from Reipoltskirchen, which is epidemiologically independent from the outbreak strains—revealed the same MLVA pattern as the Sennickerode-strains.

## Discussion

The identification of genetically closely related subpopulations of bacterial pathogens such as *F*. *tularensis* by modern genotyping methods offers the opportunity to study and describe the association between the genetic background of these subpopulations, phenotypic or ecological features like virulence, antibiotic resistance, geographic distribution or transmission patterns [6]. Several studies of *Francisella* genomics not only confirmed the current species and subspecies concept [22], but additionally revealed the association of high mortality in humans with distinct subpopulations, e.g. type A1b [23]. Analyzing the evolution and phylogeographic distribution of predominant clones in diverse endemic regions on three continents, the putative global expansion of *F*. *tularensis* ssp. *holarctica* and its complex transmission history was described [11, 24]. With the introduction of WGS based canSNPs or canINDELS assays, distinct clusters of genotypes and phylogeographic patterns were identified in Scandinavia, Georgia, Switzerland, France and other Central and Western Europe countries [11, 21, 25–28]. Despite the success of genetic studies in more diverse strain collections based on whole genome single nucleotide polymorphisms and analysis of repetitive elements [18], only few studies have reported on the diversity of *F*. *tularensis* subpopulations or clades from acute tularemia outbreaks. Besides one previous study reporting the clonal identity of 12 holarctica isolates from an epizootic event in a Phoenix [USA] zoo in 2000 [29], investigations of multiple isolates from geographically confined human outbreaks in Oulu, Finland and Ljusdal, Sweden revealed that each outbreak region was represented by just a few closely related genotypes [30]. A more comprehensive analysis using new high- resolution genotyping methods [canINDELS and MLVA] in the same endemic outbreak area and an additional emerging region in Sweden confirmed strong spatial associations between *F*. *tularensis* subpopulations and the putative areas of transmission. In some cases, infections assigned to certain single genotypes occurred exclusively in areas as small as 2 km^2^ [28], whereas in other spatially related outbreaks even different subspecies of *F*. *tularensis* have been implicated [31].

While the latter studies definitely increased our knowledge on the ecology of *F*. *tularensis*, they also disclose shortcomings of current bioinformatic and experimental procedures. WGS of one or only a few bacterial isolates were used to define a relatively small number of genetic markers, ranging from 11 to about 50 elements [SNPs, INDELS, VNTRs] which were then used to characterize up to several hundred strains [11, 25–28, 32]. Besides the inherent problem of a limited genetic resolution associated with this approach, the selection of one “representative” strain for WGS as well as the arbitrary choice of “canonical” markers may prone to bias in seldom cases caused by homoplasy or converging evolution. Additionally, the aforementioned procedure is difficult to standardize across different laboratories in diverse regions and impedes the introduction of a reliable nomenclature for identified subpopulations, clades or genotypes [6]. In view of these difficulties we decided to sequence all outbreak isolates involved in our studies and one additional reference strain [LVS] using the latest benchtop instruments [Ion Torrent PGM, Illumina MiSeq]. Sequencing of the reference strain *F*. *tularensis* ssp. *holarctica* LVS twice using different sequencing technologies led to identical results indicating that no technology bias was introduced. By defining a set of fully annotated genes present in each strain, thereby excluding repetitive elements as well as pseudogenes, we were able to compare approximately 60% of the genomes in a gene-by-gene approach [MLST^+^]. When we compared the nucleotide sequences of 1069 genes, eight outbreak isolates from the same place of transmission were completely identical demonstrating not only the very high reproducibility of the sequencing procedure but also confirming the monomorphic structure of the *F*. *tularensis* genome with pairwise average nucleotide identities [ANI] >99.2% across even subspecies [33]. The application of MLST^+^ revealed three different genotypes with different degrees of relatedness among all outbreak strains. Whereas all fatalities in the marmoset population [n = 6] as well as one isolate from a rodent trapped on the area of one facility were caused by a unique genotype belonging to the phylogenetic group B.FTNF002 [11], two distinct genotypes were shown to be responsible for the protracted outbreak in the German primate center in Goettingen.

Interestingly, both of these distinct genotypes differed from the cluster found in Sennickerode. This excludes any transmission between both animal facilities and eliminates the possibility of a common source of infection [e.g. litter or feed]. These genotypes were also representatives of two completely major subclades, B.FTNF002 and B.FSC200, respectively.

Our MLST^+^-results matched well with those obtained through current standard methods canSNP or MLVA which also showed two and three different patterns. An epidemiologically unrelated isolate, which was hitherto indistinguishable from the cluster found in Sennickerode, and which had been isolated from a human case 260 km west of Sennickerode was also analyzed. In contrast to the hierarchical SNP and MLVA typing approach, MLST^+^ was able to define a distinct genotype for this strain. This suggests that MLST^+^ might be superior to the current standard method, even when the default settings were chosen to perform the bioinformatic analyses. This algorithm enables the immediate classification of a new isolate and comparison with strains in databases. We anticipate that in combination with web-based analysis methods, it would be possible to establish a highly standardized nomenclature for subpopulations, clusters or single strains based on MLST^+^. Whereas such MLST^+^- databases have already been developed for several bacteria including pathogens [5, 34], this tool is lacking for *F*. *tularensis*. Access to a standardized, open source and curated *Francisella* database would greatly facilitate surveillance even for public health laboratories with limited technical resources because WGS may be performed by commercial services. Core genome gene SNP typing as a further analysis method did not show a higher resolution or a different conclusion for our investigated strain collection. For a more divergent subset of *Francisella tularensis*—strains, this may lead to different results, but in our case it shows, that even in spatially and temporary close area MLST^+^ may lead to a discriminatory power comparable to core genome gene SNP-typing.

For clonal species like *F*. *tularensis* it might be additionally necessary to extent the genetic analysis beyond the core genome. Resolution of the WGS analysis might be increased by incorporating accessory genome genes [already supported in SeqSphere^+^], repetitive elements [RNA coding sequences, insertion elements, VNTR, CRISPR], pseudogenes or genes of the *Francisella* pathogenicity islands. Inclusion of these elements might be mandatory for following micro-evolutionary events, which might have been missed in our current study. Unfortunately, the necessary information is not easily obtained from the raw sequence data provided by the current next-generation sequencing platforms. Because the size of some repetitive elements may exceed 2–3 kb, read lengths have to be increased and bioinformatic processing has to be improved to allow rapid *in silico* MLVA, MST or IS element typing which all have been shown to be very useful in genotyping of clonal bacterial populations [35–37].

A promising technical solution has recently been described from a new sequencing platform, PacBio RS [Pacific Biosciences, Menlo Park, CA, USA]. The PacBio produces very long reads, with average length of 4–5 kb. Such long reads will probably span most repetitive sequence elements, thus facilitating assembly and dramatically reducing the number of contigs. Shortly, it might be possible to rapidly include the complete genome information into bacterial classification. Such an approach will be necessary to follow microevolution of *F*. *tularensis* during replication in the host or in distinct enzootic cycles between mammalian hosts and blood-feeding vectors like ticks. Additionally, novel algorithms might also be needed to overcome the current limitation of resolution preventing the elucidation of the origin and temporal spread of the most recent *F*. *tularensis* clone which has emerged in France, Spain, Italy and Germany [11, 38].

## Supporting Information

S1 Data Archive Supplemental Information for MLST*-Francisella tularensis. Archive of Supplemental files are listing included and excluded targets of MLST+ analysis, overview of MLVA and canSNP results as well as nucleotide and allelic variants of all investigated strains.It also shows a comparing Minimum-Spanning Tree of MLST^+^ and wgSNP data.
(ZIP)
