# Supplementary material for: Rapid High Resolution Genotyping of Francisella tularensis by Whole Genome Sequence Comparison of Annotated Genes (“MLST+”)
Source: PLoS One. 2015 Apr 9;10(4):e0123298. doi: 10.1371/journal.pone.0123298 (PMC4391923; doi:10.1371/journal.pone.0123298)
Supplement: S1 Data Archive — Archive of Supplemental files are listing included and excluded targets of MLST+ analysis, overview of MLVA and canSNP results as well as nucleotide and allelic variants of all investigated strains.It also shows a comparing Minimum-Spanning Tree of MLST+ and wgSNP data. (ZIP) [file pone.0123298.s001.zip › Supplemental_MLSTplus_Francisella/Allele-Variants_SNPs/all_variants.pdf]

Whole Genome Sequencing
Nucleotide Variants
Cluster LVS

Target	Position	Abs. Position	LVS	F49_PGM	F49_MiSeq	Gene	Function
FTL_0146	1022	152669	G	T	T		ABC transporter ATP-binding protein

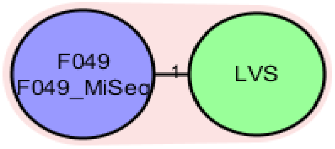

MST for 3 Samples based on 1142 columns, no missing values

Distance based on columns from F. tularensis MLST+ 1147 targets LVS:MLST+ (1142)

Comparison Table definition: F. tularensis MLST+

Comparison Table created: Jan 28, 2015 9:34 AM

Task Templates: F. tularensis MLST+ 1147 targets LVS v1.2

F. tularensis MLST+ 1147 targets LVS Cluster-Alert distance: 1

NCBI

Reference

Cluster FTNT002

Target	Position	Abs. Position	FTNF002-00	F88	F89	F90	F91	F92	F105	F112	F114	F100	F108	F109	F233	Gene	Function
FTL_0708	133	698081	G	G	G	G	G	G	G	G	G	A	A	A	G		hypothetical protein
FTL_0752	1266	744950	G	G	G	G	G	G	G	G	G	G	G	G	A		hypothetical protein
FTL_0968	120	935789	T	C	C	C	C	C	C	C	C	C	C	C	C	tyrS	tyrosyl-tRNA synthetase
FTL_1218	663	1168568	C	C	C	C	C	C	C	C	C	A	A	A	C		hypothetical protein
FTL_1282	5	1221268	C	C	C	C	C	C	C	C	C	C	C	C	A		beta-glucosidase
FTL_1534	648	1464180	C	T	T	T	T	T	T	T	T	C	C	C	C	udk	uridine kinase
FTL_1602	735	1532174	A	C	C	C	C	C	C	C	C	C	C	C	C	hemB	delta-aminolevulinic acid dehydratase
FTL_1743	159	1673175	A	C	C	C	C	C	C	C	C	C	C	C	C	xerD	integrase/recombinase

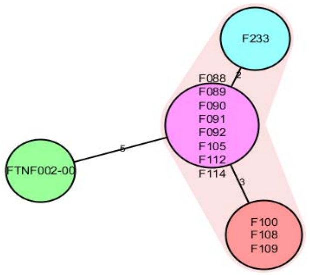

MST for 13 Samples based on 1100 columns, no missing values
Distance based on columns from F. tularensis MLST+ 1147 targets LVS:MLST+ (1100)
Comparison Table definition: F. tularensis MLST+
Comparison Table created: Jan 28, 2015 9:34 AM
Task Templates: F. tularensis MLST+ 1147 targets LVS v1.2
F. tularensis MLST+ 1147 targets LVS Cluster-Alert distance: 1

- Göttingen_2002
- NCBI
- Outgroup Sennickerode
- Sennickerode

Whole Genome Sequencing
Nucleotide Variants
Cluster FSC200

Target	Position	Abs. Position	FSC200	F101	F107	Gene	Function
FTL_0082	54	78630	C	T	T		B-type cytochrome
FTL_0287	456	273244	G	A	A		hypothetical protein
FTL_0335	108	318326	A	C	C		lipoprotein
FTL_0382	205	352713	T	C	C		amino acid permease
FTL_0556	896	538352	T	C	C		ribonuclease R
FTL_0612	924	603644	T	C	C		exopolyphosphatase
FTL_0725	480	715180	T	C	C		hypothetical protein
FTL_0766	171	755368	T	G	G	ggt	gamma-glutamyltranspeptidase
FTL_0844	219	826097	C	T	T		LysR transcriptional regulator family protein
FTL_0851	555	833113	G	A	A	rpoH	RNA polymerase factor sigma-32
FTL_0925	305	898206	T	G	G		proton-dependent oligopeptide transport (POT) family protein
FTL_0960	442	927939	T	C	C		soluble pyridine nucleotide transhydrogenase
FTL_1018	420	977706	A	C	C	serC	phosphoserine aminotransferase
FTL_1096	766	1044580	T	C	C		lipoprotein
FTL_1140	393	1082503	T	C	C		malonyl CoA-acyl carrier protein transacylase
FTL_1181	645	1131952	T	G	G	yfhQ	SpoU rRNA methylase family protein
FTL_1370	774	1302760	C	T	T		hypothetical protein
FTL_1377	154	1311261	A	T	T (warning)		hypothetical protein
FTL_1504	620	1434960	A	G	G		peroxidase/catalase
FTL_1596	30	1523075	C	A	A		hypothetical protein
FTL_1602	122	1531561	G	C	C	hemB	delta-aminolevulinic acid dehydratase
FTL_1647	861	1578787	A	G	G		major facilitator transporter
FTL_1656	86	1584636	G	A	A	uvrD	DNA helicase II
FTL_1784	1619	1716449	G	A	A	sucA	2-oxoglutarate dehydrogenase E1 component
FTL_1817	496	1750649	T	C	C	nuoN	NADH-ubiquinone oxidoreductase subunit N
FTL_1943	1683	1872762	G	A	A	priA	primosomal protein N'

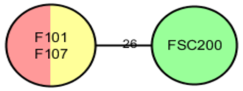

MST for 3 Samples based on 1142 columns, no missing values

Distance based on columns from F. tularensis MLST+ 1147 targets LVS:MLST+ (1142)

Comparison Table definition: F. tularensis MLST+

Comparison Table created: Jan 28, 2015 9:34 AM

Task Templates: F. tularensis MLST+ 1147 targets LVS v1.2

F. tularensis MLST+ 1147 targets LVS Cluster-Alert distance: 1

Göttingen_2002

Göttingen_2005

NCBI
