# Supplementary figures and images for: Rapid High Resolution Genotyping of Francisella tularensis by Whole Genome Sequence Comparison of Annotated Genes (“MLST+”)
Source: PLoS One. 2015 Apr 9;10(4):e0123298. doi: 10.1371/journal.pone.0123298 (PMC4391923; doi:10.1371/journal.pone.0123298)

A

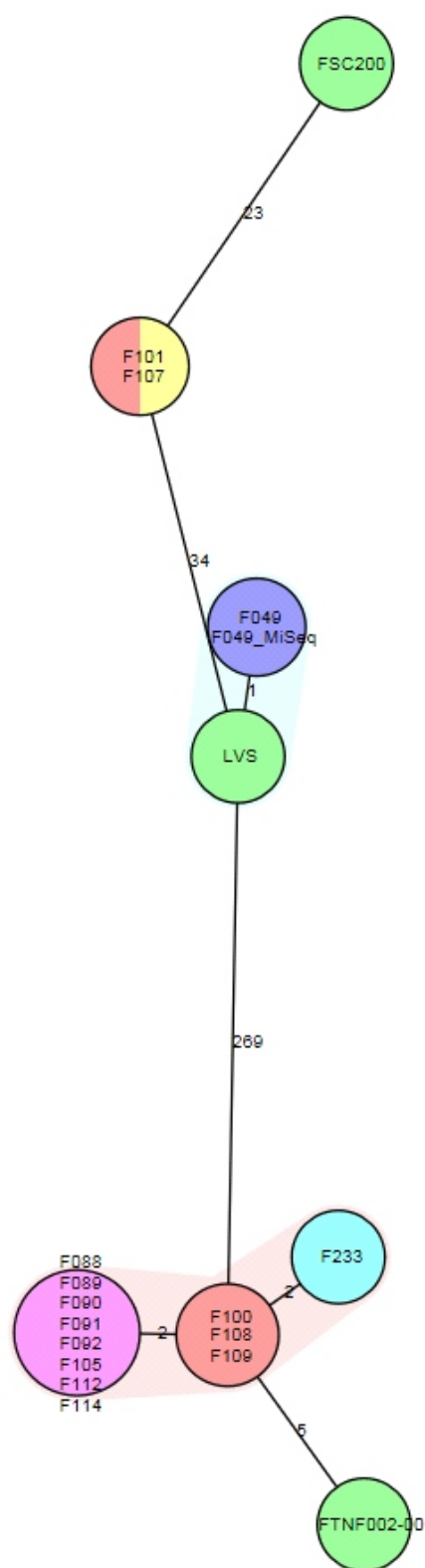

B

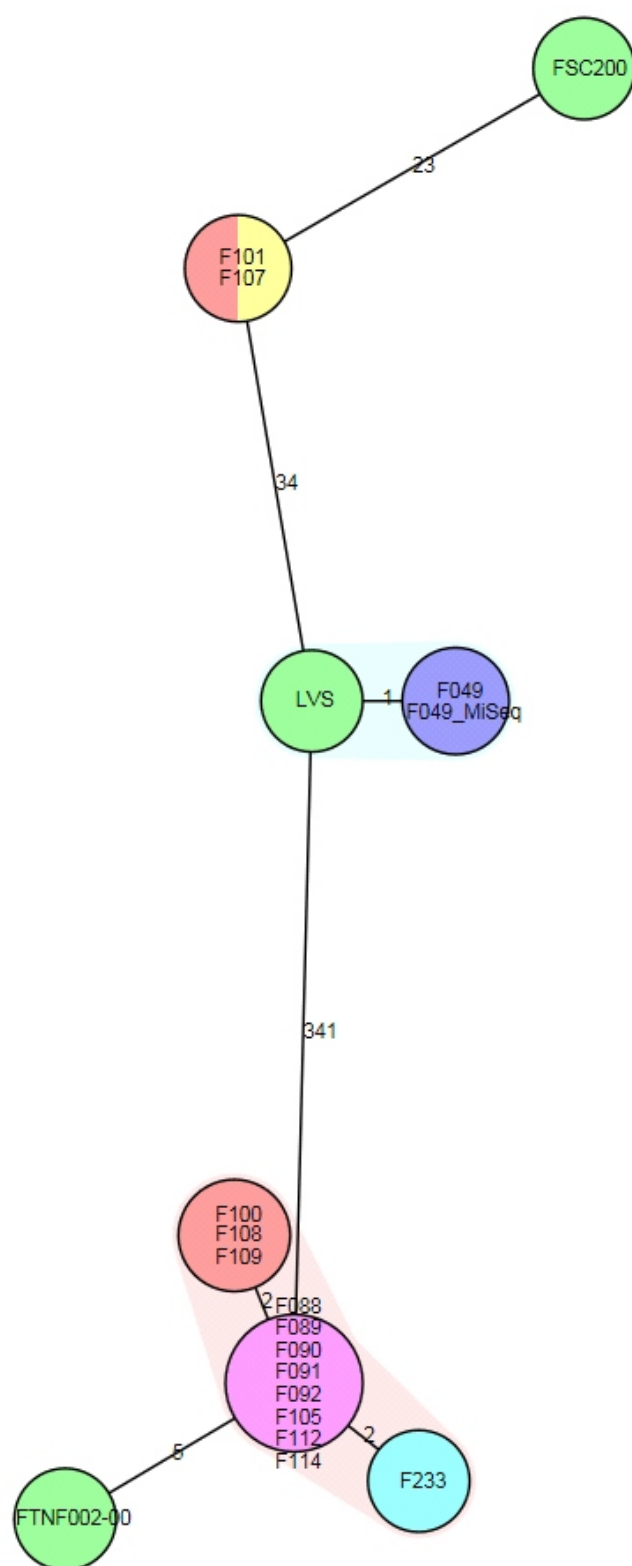

Minimum Spanning Tree based on  
A) MLST<sup>+</sup>- Allele-Codes and B) wgSNPs

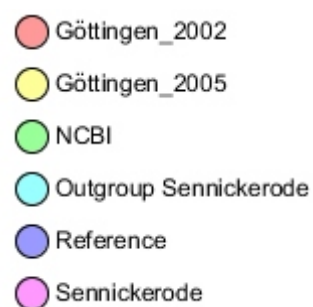

Supplement: S1 Data Archive — Archive of Supplemental files are listing included and excluded targets of MLST+ analysis, overview of MLVA and canSNP results as well as nucleotide and allelic variants of all investigated strains.It also shows a comparing Minimum-Spanning Tree of MLST+ and wgSNP data. (ZIP) [file pone.0123298.s001.zip › Supplemental_MLSTplus_Francisella/Allele-Variants_SNPs/Suppl_Figure_MST_wgSNPvsMLSTplus.pdf]
